# Effect of personalized moderate exercise training on Wistar rats fed with a fructose enriched water

**DOI:** 10.1186/s12986-018-0307-6

**Published:** 2018-10-03

**Authors:** Julie Dupas, Annie Feray, Anthony Guernec, Morgane Pengam, Manon Inizan, François Guerrero, Jacques Mansourati, Christelle Goanvec

**Affiliations:** 1 EA 4324: Optimisation des Régulations Physiologiques, Université de Bretagne Occidentale, 6 avenue Le Gorgeu, 29238 Brest Cedex 3, France; 2 UFR Sciences et Techniques, Université de Bretagne Occidentale, 6 avenue Le Gorgeu, 29237 Brest Cedex 3, France; 3 UFR Sciences du Sport et de l’Education, 20 avenue Le Gorgeu, 29238 Brest Cedex 3, France; 4 Département de Cardiologie, Centre Hospitalo-Universitaire de Brest, Boulevard Tanguy Prigent, 29200 Brest, France; 5 IBSAM: Institut Brestois Santé Agro Matière, UFR Médecine, avenue Camille Desmoulin, 29200 Brest, France

**Keywords:** Metabolic syndrome, Fructose enriched water rat model, Aerobic training

## Abstract

**Background:**

Metabolic Syndrom has become a public health problem. It mainly results from the increased consumption of fat and sugar. In this context, the benefits of personalized moderate exercise training were investigated on a metabolic syndrome male wistar rat model food with fructose drinking water (20–25% *w*/*v*). Different markers including body weight, metabolic measurements, blood biochemistry related to metabolic syndrome complications have been evaluated.

**Methods:**

Male Wistar rats were randomly allocated to 4 groups: control (sedentary (C, *n* = 8) and exercise trained (Ex, n = 8)), fructose fed (sedentary (FF, n = 8) and exercise trained fructose fed rats (ExFF, *n* = 10)). ExFF and Ex rats were trained at moderate intensity during the last 6 weeks of the 12 weeks-long protocol of fructose enriched water. Metabolic control was determined by measuring body weight, fasting blood glucose, HOMA 2-IR, HIRI, MISI, leptin, adiponectin, triglyceridemia and hepatic dysfunction.

**Results:**

After 12 weeks of fructose enriched diet, rats displayed on elevated fasting glycaemia and insulin resistance. A reduced food intake, as well as increased body weight, total calorie intake and heart weight were also observed in FF group. Concerning biochemical markers, theoretical creatinine clearance, TG levels and ASAT/ALAT *ratio* were also affected, without hepatic steatosis. Six weeks of 300 min/week of moderate exercise training have significantly improved overweight, fasting glycaemia, HOMA 2-IR, MISI without modify HIRI. Exercise also decreased the plasma levels of leptin, adiponectin and the ratio leptin/adiponectin. Regarding liver function and dyslipidemia, the results were less clear as the effects of exercise and fructose-enriched water interact together, and, sometimes counteract each other.

**Conclusion:**

Our results indicated that positive health effects were achieved through a personalized moderate training of 300 min per week (1 h/day and 5 days/week) for 6 weeks. Therefore, regular practice of aerobic physical exercise is an essential triggering factor to attenuate MetS disorders induced by excessive fructose consumption.

## Background

Metabolic syndrome (MetS) is a cluster of different symptoms including, for example insulin resistance (IR), abdominal obesity, dyslipidemia, hypertension [1] and is strongly related to the development of Type 2 Diabetes (T2D) increasing the risk by 5-fold [2].The fast increase in MetS prevalence is strongly related to changes of our lifestyle characterized nowadays by reduced physical activities and bad habits in our diet [3]. Particularly, high consumption of sugar [4], specifically fructose used in soft drinks as a common sweetener, contributed to the development of MetS [5].

Although they have not always expressed all signs of the syndrome, as shown by previous studies [6, 7], rats fed this type of diet have been used as an experimental model of human MetS [8]. Especially, fructose overload in drinking water can induce MetS in rats as demonstrated previously [9, 10]. Our group had validated a rat model of MetS without obesity after 21 weeks of fructose overload. This model is close to what happens in human in term of risk and pathology development [11]. Its originality consists in the development of cardiovascular risk factors at early age after consumption of a real-like fructose enriched diet at similar levels to those children could be exposed. Otherwise, we have also shown an overweight after 12 weeks of the same fructose overload [12]. Consequently, new markers must be studied to identify the origin of this overweight. Adipokines are reportedly associated with the risk for MetS, T2D and cardiovascular diseases. Among them, leptin and adiponectin may be risk markers of fat-induced dyslipidemia and IR. Their plasma levels are directly proportional to secretion rates of the adipokine by adipose tissue [13]. Persons at high risk seemingly have high levels of plasma leptin and low levels of plasma adiponectin. Because leptin and adiponectin have opposite effects, the LAR (Leptin/Adiponectin ratio) has been proposed as a better biomarker for IR [14, 15] and MetS diagnosis criteria [16, 17] than leptin and adiponectin separately.

In the context of MetS, non-pharmacological and non-invasive therapeutic benefits, such as exercise training (ET), are becoming increasingly acknowledged. To modify lifestyle and reduce risk factors associated with MetS, ET may be implemented since it has been shown to fight the negative effects of excess fructose intake [18–21]. Indeed, chronic ET, over 4 weeks long [22], has beneficial impact on some cardiovascular risk factors like hyperglycaemia, obesity and hypertension, and is now commonly used in cardiovascular disease primary and secondary prevention [23]. For most adults suffering with diabetes, the updated recommendations of the American Diabetes Association [24] propose doing 150 min or more of moderate-to-vigorous intensity activity weekly, spread over at least 3 days/week, with no more than 2 consecutive days without activity. Training at moderate intensity (50 to 70% of maximal aerobic capacity) seems to be the best compromise between efficiency and feasibility for individual care [25]. Recently, Bird and colleagues (2017) [26] have reported that: “the potential to adapt and improve insulinosensitivity is likely to be influenced by the basal state of the participants: with healthy participants, overweight/obese, prediabetic MetS, and patients with diabetes all likely to differ in the magnitude of adaptation and improvement”. As a consequence, a personalized aerobic moderate ET (70% of maximal aerobic speed (MAS)) of 300 min/week has been chosen. Although the importance of taking gender into account is an evidence, in the present paper, we used only male to avoid estrogen interaction during our investigations of the training effects on MetS [27]. Using our non-obese model of MetS male Wistar rats, this integrative study aimed to evaluate the effects of personalized protocol training on several metabolic markers of the MetS: body weight, blood biochemistry especially triglyceridemia, glucose homeostasis, IR and hepatic dysfunction.

## Methods

### Animals

All experiments were approved by local French ethical comity (CEFEA, n°74), and authorized by the French “Ministère de l’Éducation Nationale, de l’Enseignement Supérieur et de la Recherche” under the number 2269.

Fifty male Wistar rats (weight < 49 g, Janvier labs, Le Genest Saint Ile, France), were housed individually at the age of three weeks in a light (12 h:12 h light/dark cycle) and temperature (21 °C ± 1 °C) controlled animal facility. Rats were all fed with a standard diet (Kliba Nafag, M/R Maintenance, 3.152 kcal/g). Rats were randomly assigned to one of the two groups: control rats with tap water (C, *n* = 24) and fructose fed rats (FF, *n* = 26) with fructose (4 kcal/g) enriched drink (20% *w*/*v* from age 3 weeks to age 9 weeks) for 6 weeks. To test training effect on fructose fed animals, each group of rats was divided in 2 at the age of 9 weeks. We obtained 4 groups: control rats (sedentary (C, *n* = 8) and exercise trained (Ex, n = 8)) with tap water, fructose fed rats (sedentary (FF, n = 8) and exercise trained fructose fed rats (ExFF, *n* = 10)) with fructose enriched drink (25% *w*/*v* from age 10 weeks to age 15 weeks). ExFF and Ex rats were trained at moderate intensity for 6 weeks. The other rats (*n* = 16) were sacrificed at the age of 9 weeks and were used to define our model [12]. Fructose enriched drinks were changed every couple of days; water bottles were sterilized every week. Body weight (bw) was measured weekly. Food, drink and total calorie intakes rat were recorded individually once a week. The consumption was estimated as daily for 100 g of rat. The three parameters were expressed as Area Under the Curve (AUC), calculated by trapezoidal method.

### Personalized moderate exercise protocol

Prior to the moderate exercise protocol, all rats underwent an evaluation of their maximal aerobic speed (MAS). The protocol consisted of an exercise session where the speed was progressively incremented by 3.33 m/min for five minutes and then by 1.6 m/min until rats were unable to run anymore [27, 28]. This last speed was used as their MAS. After the first MAS evaluation, rats were randomly divided into the four groups defined before: sedentary C, FF and trained groups Ex, ExFF. MAS was re-evaluated twice during the training period in order to adapt training intensity and to evaluate exercise efficiency only on trained group to avoid acute effect of MAS on sedentary group. Each trained rats (Ex and ExFF) ran on a treadmill 5 days/week for 1 h at a speed equivalent to 70 ± 5% of their MAS, while FF and C remained on the turned-off treadmill.

### Sampling

Rats were anesthetised with Ketamine (Ketamine 100, Virbac, 80 mg/kg) and Xylazine (Rompun 2%, Bayer, 12 mg/kg) injected intramuscularly (into the left back leg). Blood was collected intraventricularly. Blood was collected into 2 mL sampling tube (pre-coated with EDTA). Plasma was obtained after centrifugation for 15 min at 1000 g. Heart was collected and cleared of residual blood and then weighted. Plasma and organs were immediately frozen in liquid nitrogen and then stored at − 80 °C for further analysis.

Heart weight (% bw) was calculated as followed:

$$ \mathrm{Heart}\ \mathrm{weight}\ \left(\%\mathrm{bw}\right)=\frac{\mathrm{heart}\ \mathrm{weight}(g)}{\mathrm{body}\ \mathrm{weight}(g)}\times 100 $$

### Metabolic measurements

After a 15 h without food, fasting glucose was measured in blood collected by a single prick onto the mandibular veins (allowing only one drop to come off) using a glucometer (Accu-Chek Performa, Roche, Meylan, France) [29]. Oral Glucose Tolerance Test (OGTT) was performed at the age of 13 weeks and was described previously [11]. Plasma from OGTT blood sample was obtained after centrifugation at 2000 g for 5 min. Plasma was then frozen and stored at − 80 °C before further analysis. Insulin concentration was evaluated on those plasma samples using ELISA methods (Rat Insulin Elisa, ALPCO, Eurobio, Courtaboeuf, France). Glucose and Insulin responses were expressed as AUC, calculated by trapezoidal method, and Net AUC calculated after subtracting the baseline concentration.

Based on OGTT and insulin concentrations results, three indicators of insulin resistance and sensitivity were determined: the Homeostatic Model Assessment for Insulin Resistance (HOMA 2-IR), the Hepatic Insulin Resistance Index (HIRI) and the Muscle Insulin Sensitivity Index (MISI). HOMA 2-IR was calculated using a HOMA 2 IR calculator software [30] (software available at https://www.dtu.ox.ac.uk/homacalculator, Oxford University) and using OGTT data, i.e. fasting insulinemia and fasting glycaemia were taken at *t* = 0 min (before the ingestion of a high dose of glucose (1 g/kg bw). HIRI is the product of the net AUC for blood glucose multiplied by the net AUC for plasma insulin during the first 30 min of the OGTT. MISI is the rate of blood glucose concentration decay from its peak value to its nadir divided by the mean plasma insulin concentration during the OGTT [31, 32].

Adiponectin and leptin concentrations were evaluated on plasma samples using ELISA methods (Rat Adiponectin Elisa, ALPCO, and “Mouse and Rat Leptin ELISA” BioVendor®, Eurobio, Courtaboeuf, France). The LAR was then calculated.

### Blood biochemistry

Blood chemistry measurements were done on a Koné lab 20 (Thermo Scientific) using adapted kit for: ASpartate AminoTransferase activity (ASAT)(Biomerieux), ALanine AminoTransferase activity (ALAT) (Biomerieux), Creatinine (Jaffé method, Fisher Brahms), Albumin (Bromocresol green method, Biomerieux), Non-Esterified Fatty Acid (NEFA) (Wako), TriGlycerides (TG) (PAP methods, Biomerieux), total cholesterol (Cholesterol RTU, Biomerieux). The ASAT/ALAT ratio was then calculated. Different molar ratios were calculated as a part of the lipids levels evaluation: NEFA to cholesterol ratio [33], NEFA to Albumin ratio [34], and cholesterol to TG ratio [35]. Theoretical creatinine clearance was calculated using the Cockcroft and Gault formula [36], that has been already used in rat model [37].

$$ \mathrm{Theoretical}\ \mathrm{creatinine}\ \mathrm{clearance}\;\left(\frac{\mathrm{mL}}{\min}\right)=\kern0.5em \frac{{\left(140\hbox{-} \mathrm{age}\kern0.5em \left(\mathrm{years}\right)\right)}^{\ast}\;\mathrm{body}\kern0.5em \mathrm{weight}\;\left(\mathrm{kg}\right)}{\left(\mathrm{creatinine}\;\left(\frac{\mathrm{mg}}{\mathrm{dL}}\right)\ast 72\right)} $$

### Citrate synthase (CS) activity

50 mg of either left ventricle or left soleus were homogenised in a 4 °C Tris HCl buffer (0.1 M, pH 8.1) with a Polytron. The homogenate was then collected and used immediately for analysis. Measurements of CS activity were done by an indirect method [28] using 5,5-dithio-bis-2-nitrobenzoic acid (DTNB). CS activity was thus measured at 412 nm (Evolution 201, Thermo-Scientific).

### Hepatic histology

Small portions of the liver were sampled and immediately put in fixative solution (Bouin) for at least 48 h. Samples were then embedded in paraffin and transverse slices of 5 μm were cut and stained by eosin/hematoxylin to detect eventual steatosis. Classification of steatosis evolution was determined as followed: score 0: no lipid droplet, score 1: less than 10 microvesicles of lipid droplets, score 2: more than 10 micro-vesicles lipid droplets, score 3: macro and micro vesicles lipid droplets > 30, score 4: steatosis.

### Statistics

All results are expressed as means ± standard error of mean (SEM). All statistics were performed using Statistica v. 12 software (StatSoft, France). Normality of population was tested using the Shapiro-Wilk test. Adapted tests were then performed (Kruskal-Wallis, Mann and Whitney U test, two-way analyses of variance (ANOVA) and ANOVA for repeated measures). ANOVA were followed by a post-hoc test (HSD for n different). Significant differences (*p* < 0.05) between groups were mentioned by different letters (a, b, c…) in the tables and figures.

## Results

### Personalized moderate exercise protocol

In order to evaluate exercise training efficiency, CS activity levels were measured in both soleus muscle and myocardium of left ventricle as a marker of mitochondrial content in the 4 experimental groups (Table 1). In both tissues, ET increased CS activity (*p* < 0.01) while fructose enriched diet decreased it (*p* < 0.05). There was also an interaction between both of these two factors (*p* < 0.001). The Ex soleus muscles have a higher CS activity than C (55.6 ± 3.3 vs. 35.6 ± 2.7 μmol/min/mg FW), likewise ExFF soleus muscles compared to FF (36.6 ± 1.8 vs. 28.1 ± 1.9 μmol/min/mg FW). Finally, the hearts of Ex rats displayed a greater CS activity than C (184.0 ± 3.4 vs. 98.2 ± 10.2 μmol/min/mg FW).

**Table 1:** Citrate synthase activity in two different tissues at 15 weeks

	Muscle	C (n = 8)	Ex (n = 8)	FF (n = 8)	ExFF (*n* = 10)
CS activity (μmol/min/mg FW)	Soleus	35.6 ± 2.7a	55.6 ± 3.3b	28.1 ± 1.9 c	36.6 ± 1.8 a
	Left ventricle	98.2 ± 10.2a	184.0 ± 3.4b	116.1 ± 3.4 ac	135.4 ± 4.3 c

Values are means ± SEM in μmol/min/mg of Fresh Weight (FW). For the soleus and the left ventricle, training effect (*p* < 0.01), fructose effect (*p* < 0.05), interaction (*p* < 0.001) were statistically significant. Statistical differences with post-hoc test are observed when lowercase letters (a, b, c) under values are different with *p* < 0.05

MAS was measured regularly in trained animals (Ex, ExFF) both to evaluate ET efficiency as well as to normalize individual training. Results are summarized in Table 2. Before the start of the treadmill training, both groups (Ex and ExFF, age: 9 weeks) have similar MAS values: 31.00 ± 0.67 and 29.50 ± 0.83 m/min, respectively. Inside Ex group, MAS increases continuously at the age of 12 and 14 weeks, respectively after 3 and 5 weeks of ET (*p* < 0.001) compared to the previous MAS. It is the same in ExFF group. It can also be noticed that, soon as after 3 weeks of training, Ex displayed a higher MAS than ExFF (Ex: 41.83 ± 1.67 vs. ExFF: 34.33 ± 0.67 m/min, *p* < 0.001). The same conclusion can be drawn after 5 weeks of ET (Ex: 47.33 ± 2.00 vs. ExFF: 38.67 ± 0.15 m/min, *p* < 0.001).

**Table 2:** Effect of moderate training on MAS (m/min)

Age	Ex (n = 8)	ExFF (*n* = 8)	Ex vs ExFF
9 weeks	31.00 ± 0.67 a	29.50 ± 0.83 a	NS
12 weeks	41.83 ± 1.67 b	34.33 ± 0.67 d	p < 0.001
14 weeks	47.33 ± 2.00 c	38.67 ± 0.15 e	p < 0.001

Values are means ± SEM. Statistical differences with post-hoc test are observed between groups. Different lowercase letters (a, b, c, d, e under values) indicate time effect in each experimental group. Difference between groups at each age appears as *p* value. NS: Non Significant

### Morphometric and metabolic measurements

**Fig. 1:**
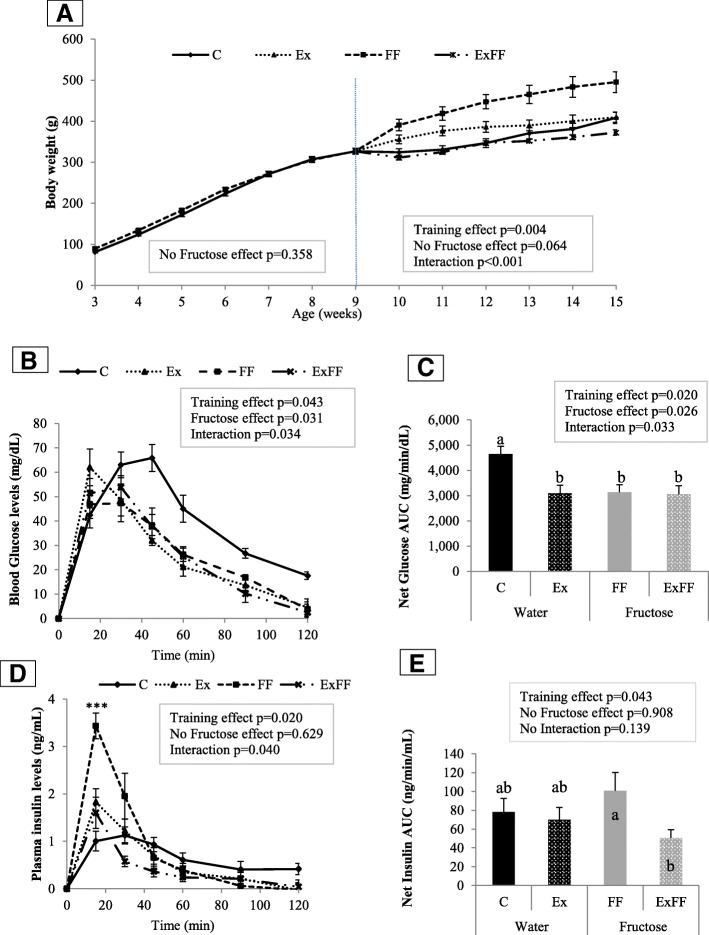
Weight, glucose and insulin tolerance measured during the study. **a** – Body weight evolution for the whole experiment. **c** control (*n* = 24 for 3 to 9 weeks, and *n* = 8 for 9 to 15 weeks). Ex: Exercise standard diet (n = 8). FF: fructose supplemented (*n* = 26 for 3 to 9 weeks and n = 8 for 9 to 15 weeks). ExFF: Exercise fructose supplemented (*n* = 10). **b** - Effect of training and fructose supplementation on blood glucose levels during OGTT, 15, 30, 45, 60, 90, 120 min after glucose charge in all groups, after subtracting baseline concentrations at 13 weeks old. **c** - Evolution of net glucose AUC in the four rat groups at 13 weeks old. **d** - Effect of training and fructose supplementation on plasma insulin levels during OGTT after subtracting baseline concentrations at 13 weeks old.*C compared to FF (***p < 0.001). **e** - Evolution of net insulin AUC in the four rat groups at 13 weeks old. For **a**, **b**, **c**, **d**, **e** figures: groups C: control (*n* = 7). Ex: Exercise standard diet (*n* = 8). FF: fructose supplemented (n = 7). ExFF: Exercise fructose supplemented (*n* = 10). Values are means ± SEM. For C and E figures, statistical differences with post-hoc test are observed when lowercase letters (**a**, **b**) are different with *p* < 0.05

The Fig. 1a displays the body weight evolution of experimental groups. During the six first weeks, the body weight was similar between C and FF. After week 9, training effect was significant (*p* = 0.004) and interaction (fructose effect x training) was observed (*p* < 0.001). Post-Hoc test displayed higher weights in FF group compared with the C group and ExFF group. At 15 weeks (Table 3), larger body weight was observed only in FF group. For the results on heart weight after 15 weeks of the experiment (Table 3), only FF heart weight was bigger than C (0.32 ± 0.010 vs. 0.27 ± 0.02% bw).

**Table 3:** Effects of training and fructose supplementation on morphological and metabolic characteristics of rats

	C	Ex	FF	ExFF	ANOVA2 or Kruskall Wallis
Body weight at 15 weeks (g)	408.50 ± 14.07 a (n = 8)	409.63 ± 12.14 a (n = 8)	495.25 ± 25.26 b (n = 8)	372.50 ± 6.47 a (n = 10)	Training effect *p* = 0.016
Heart weight (% bw)	0.27 ± 0.02 a (n = 8)	0.30 ± 0.01 ab (n = 8)	0.32 ± 0.01 b (n = 8)	0.29 ± 0.01 ab (n = 10)	Interaction *p* = 0.007
Food intake AUC for 6 weeks (g/day/100 g)	41.26 ± 0.99 a (n = 8)	37.74 ± 1.02 b (n = 8)	30.48 ± 0.73 c (n = 8)	29.53 ± 0.57 c (n = 10)	Training effect *p* = 0.02 Fructose effect *p* < 0.001
Drink intake AUC for 6 weeks (mL/day/100 g)	74.10 ± 3.22 a (n = 8)	54.44 ± 3.90 b (n = 8)	81.14 ± 1.87 a (n = 8)	80.27 ± 3.43 a (n = 10)	Training effect *p* = 0.004 Fructose effect *p* < 0.001 Interaction *p* = 0.007
Total Calorie intake AUC for 6 weeks (kcal/day/100 g)	130.06 ± 3.11 a (n = 8)	118.96 ± 3.21 a (n = 8)	177.20 ± 1.62 b (n = 8)	173.34 ± 3.75 b (n = 10)	Fructose effect *p* < 0.001
Fasting glycaemia (mg/dL)	88.14 ± 3.05 a (n = 7)	77.38 ± 3.41 b (n = 8)	98.29 ± 3.01 a (n = 7)	86.70 ± 2.95 a (n = 10)	Training effect p = 0.002 Fructose effect *p* = 0.006
HOMA 2-IR	1.62 ± 0.16 ab (*n* = 6)	0.74 ± 0.40 a (n = 8)	2.87 ± 0.43 b (n = 7)	1.48 ± 0.34 ab (n = 10)	Training effect *p* = 0.002 Fructose effect *p* = 0.027
HIRI (×10^4^)	2.61 ± 0.40 (n = 7)	4.74 ± 0.80 (n = 8)	7.27 ± 1.23 (*n* = 7)	3.67 ± 0.90 (n = 10)	No effect
MISI	0.50 ± 0.04 ab (n = 7)	0.75 ± 0.11 b (n = 8)	0.34 ± 0.04 a (n = 7)	0.77 ± 0.07 b (n = 10)	Training effect *p* < 0.001
Fasting Leptin (ng/mL)	4.52 ± 0.40 a (n = 6)	1.38 ± 0.18 b (n = 8)	4.20 ± 0.92 a (n = 8)	1.92 ± 0.27 c (*n* = 9)	Training effect *p* < 0.001
Fasting Adiponectin (μg/mL)	10.81 ± 0.58 a (n = 8)	8.26 ± 1.06 ab (n = 6)	9.31 ± 0.61 ab (n = 8)	7.79 ± 0.45 b (n = 8)	Training effect *p* = 0.005
Ratio leptin/adiponectin (LAR)	0.46 ± 0.06 a (n = 6)	0.19 ± 0.03 b (n = 6)	0.43 ± 0.07 a (n = 8)	0.27 ± 0.04 ab (n = 8)	Training effect *p* = 0.0004

Values are means ± SEM. The results of ANOVA 2 or Kruskal Wallis were displayed in the extreme right column. Statistical differences with post-hoc test are observed when lowercase letters (a, b, c under values) are different with *p* < 0.05

Anova 2 analysis of OGTT curves (Fig. 1b) and net glucose AUC (Fig. 1c) at 13 weeks old, showed that the fructose enriched diet had an effect on glucose levels (*p* = 0.031 and *p* = 0.026 respectively), and ET have had an analog effect (*p* = 0.043 and *p* = 0.020 respectively). Surprisingly, post-hoc test displayed higher net glucose AUC for C group compared to the others (*p* < 0.05).

Insulin levels were measured concomitantly to glucose levels during OGTT. Results are displayed in Fig. 1d and e. Differences could be found between groups both in term of the entire curve (*p* < 0.05) as well as at specific time. Indeed, as a whole, FF had higher insulin levels than C (respectively *p* < 0.01). In a more precise manner, 15 min after the beginning of OGTT, FF have higher insulin levels than the others groups (*p* < 0.001). For net insulin AUC (Fig. 1e) only training induces a decrease (p = 0.043).

Table 3 also summarizes morphological and metabolic markers of 15 weeks old rats. Body weight was significantly higher in FF group (*p* < 0.05). Heart weight for FF compared to C was upper (p < 0.05). Food intake AUC was significantly lower in FF group compared to C group without modification of drink intake while total calorie intake was increased. Training protocol in ExFF did not changed food, drink and total calorie intakes AUC compared to FF. The fructose enriched diet increased fasting glycaemia (*p* < 0.05). ET reduced fasting glycaemia (*p* < 0.05), moreover, there was no interaction between the effects of diet and exercise. From glucose and insulin levels during OGTT, three markers were calculated: HOMA 2-IR, HIRI and MISI (Table 3). With regard to HOMA 2-IR, it can be noticed that fructose diet increasing it (*p* = 0.027) while ET lowering it (*p* = 0.002). This marker indicates IR for a value higher than 1.85 [38]: only FF group displayed a higher value (2.87 ± 0.43). For HIRI, neither fructose, nor training provided significant impact and only ET increased MISI (*p* > 0.001). Concerning leptin, adiponectin and LAR, fructose displayed no effect and exercise decreased their plasma levels (*p* < 0.01).

### Blood biochemistry

Table 4 shows the results of blood biochemistry analysis. The theoretical creatinine clearance has been studied: this value was modified by both fructose enriched diet and ET (both *p* < 0.05). The other values shown in Table 4 concerning the lipid profile. First, NEFA, total cholesterol and TG were directly measured. Interaction between training and fructose enriched diet displayed a significant effect on total cholesterol level (*p* < 0.001). TG level was only modified by fructose supplementation (*p* = 0.025). Three molar ratios were then calculated: NEFA/Cholesterol, NEFA/Albumin and total cholesterol/TG, results are shown in Table 4. NEFA/Cholesterol and NEFA/Albumin results showed no difference between groups. Using an ANOVA 2, the ratio total cholesterol/TG was surprisingly decreased by the fructose enriched diet (*p* = 0.04), an interaction between the effects of the diet and ET were also found (p = 0.02). Indeed, Ex had a higher total cholesterol/TG ratio than C and ExFF (respectively 4.27 ± 0.69 vs. 2.06 ± 0.27 and 1.88 ± 0.55).

**Table 4:** Effects of moderate exercise and fructose supplementation on biochemical blood markers

	C	Ex	FF	ExFF	ANOVA2 or Kruskall Wallis
Theoretical creatinine clearance (ml/min)	1.30 ± 0.10 ab (n = 8)	1.14 ± 0.05 a (n = 8)	1.53 ± 0.08 b (n = 8)	1.22 ± 0.04 a (n = 9)	Training effect *p* = 0.002 Fructose effect *p* = 0.037
NEFA (μmol/L)	382.78 ± 32.47 (n = 8)	403.38 ± 41.70 (n = 8)	444.21 ± 26.08 (n = 8)	386.52 ± 25.99 (n = 9)	No effect
Total cholesterol (mg/L)	629.16 ± 35.75 a (n = 8)	851.07 ± 30.83 b (n = 8)	713.97 ± 54.11 ab (n = 8)	599.75 ± 38.93 a (n = 9)	Interaction *p* < 0.001
Triglycerides (mg/L)	769.35 ± 89.67 ab (n = 8)	530.02 ± 72.47 a (n = 8)	941.39 ± 202.35 ab (n = 8)	1058.82 ± 175.57b (n = 9)	Fructose effect *p* = 0.025
NEFA/Choles-terol (molar ratio)	0.24 ± 0.02 (n = 8)	0.18 ± 0.02 (n = 8)	0.23 ± 0.02 (n = 8)	0.26 ± 0.03 (n = 9)	No effect
NEFA/Albumin (molar ratio)	0.73 ± 0.06 (n = 8)	0.72 ± 0.07 (n = 8)	0.87 ± 0.04 (n = 8)	0.66 ± 0.05 (n = 9)	No effect
Cholesterol/Tri-glycerides (molar ratio)	2.06 ± 0.27 a (n = 8)	4.27 ± 0.69 b (n = 8)	2.23 ± 0.43 a (n = 8)	1.88 ± 0.55 a (n = 9)	Fructose effect *p* = 0.04 Interaction *p* = 0.02

Values are means ± SEM. The results of ANOVA 2 or Kruskal Wallis are displayed in the extreme right column. Statistical differences with post-hoc test are observed when lowercase letters (a, b, under values) are different (*p* < 0.05)

### Hepatic histology

**Fig. 2:**
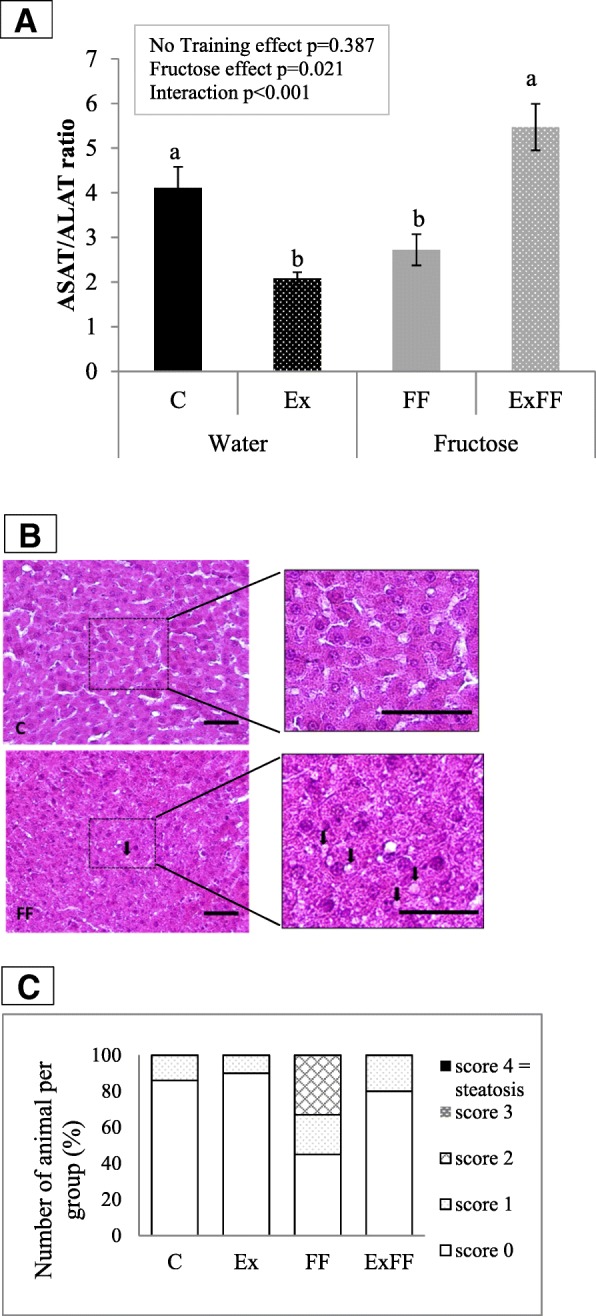
Evolution of hepatic complications. **a** – Quantification of ASAT/ALAT Ratio. C: control (*n* = 7). Ex: Exercise standard diet (n = 7). FF: fructose supplemented (*n* = 8). ExFF: Exercise fructose supplemented (n = 8). Values are means ± SEM. Statistical differences are observed when lowercase letters (**a**, **b**) are different. **b** - Hematoxylin and Eosin staining of liver (× 40 and × 100) for one rat of C group and one rat of FF group. Bar scale =50 μm. Arrow = lipid droplet. Small lipid droplets can be found only on FF group. **c** – Classification of steatosis evolution. The scale was determined as: Score 0: no lipid droplet. Score 1: less than 10 microvesicles of lipid droplets. Score 2: more than 10 microvesicles lipid droplets. Score 3: macro and micro vesicles lipid droplets > 30. Score 4: steatosis. The observation was performed on slides of each rat of C (n = 8), Ex (n = 8), FF (n = 8), ExFF (*n* = 10). The results displayed the percentage of rat for each score

The ASAT/ALAT ratio (Fig. 2a) was significantly modified by the fructose enriched diet (*p* = 0.021). An interaction between the diet and ET (*p* < 0.001) was also found by an ANOVA 2 analysis. Indeed ExFF had a higher ASAT/ALAT ratio than both FF and Ex (respectively 5.47 ± 0.52 vs. 2.72 ± 0.35, p < 0.001; 5.47 ± 0.52 vs. 2.08 ± 0.14, p < 0.001) but Ex had a lower ratio than C (2.08 ± 0.14 vs. 4.11 ± 0.47, *p* < 0.01). Figures 2b and c suggested the presence of microvesicles of lipid droplets only in the FF group with a score of 2 (about 33% of rat observed). Whatever the group, no steatosis could be observed.

## Discussion

The aim of this study was to evaluate the effects of personalized moderate ET in a rat model of fructose supplementation just after weaning, on various parameters including metabolic measurements and blood biochemistry.

Previous studies have shown that a diet enriched in fructose (20–25% *w*/*v* in drinking water for 12 weeks) induced early stage of T2D without inducing obesity in Wistar rats [12]. With the same supplementation for 21-week-long, this model induced a MetS without steatosis and not always a T2D and obesity [11]. This study is consistent with our previous findings regarding the fructose enriched diet. Indeed, after 12 weeks of fructose enriched diet, rats developed elevated fasting glycaemia (Table 3) and IR (shown with HOMA 2-IR > 1.85), an increased body weight (provided by increased total calorie intake) and heart weight only in FF group (Table 3). Concerning biochemical markers, theoretical creatinine clearance, TG levels (Table 4) and ASAT/ALAT *ratio* (Fig. 2a) is also affected, without hepatic steatosis (Fig. 2b, c).

In our study, we first evaluated the efficiency of our personalized ET using the CS activity as a marker of mitochondrial content [28, 39]. For the two muscles studied, the training protocol has improved CS activity. That is consistent with previous finding [40] and prove the efficiency of our training protocol (Table 1). Furthermore, MAS (Table 2) was enhanced at each measurement, regardless of the diet, another evidence of our training protocol efficiency [28]. It is to notice that performance was more increased in control rats (Ex group: + 52%) than in fructose-supplemented animals (ExFF: + 31%, *p* < 0.001). The effect of personalized moderate ET on pour model can thus be fully discussed.

First, MetS markers (fasting glycaemia, HOMA 2-IR, HIRI, MISI as well as OGTT curves, net glucose AUC, plasma insulin levels, net insulin AUC) were studied. ET helps to reduce fasting glycaemia, insulin resistance (HOMA 2-IR lowered below 1.85) and improves muscular insulin sensitivity (MISI) without modify hepatic resistance (HIRI) (Table 3). ET restores the blood glucose levels (in OGTT curves). The concomitantly measured insulin levels showed interesting results (Fig. 1 d). Indeed, ET reduces insulin levels in ExFF group, thus showing the positive impact of exercise on glucose metabolism in MetS rats likewise others authors [19, 20, 41, 42]. About the net glucose AUC, our results are not obvious: training seems to restore Impaired Glucose Tolerance (IGT). Surprisingly, our results looks like C group impaired glucose tolerance. In this paper, net glucose AUC is used (and not total AUC like for Isi-Gly calculator) to determine HIRI and MISI, markers accurately than Isi-Gly to evaluate ET effect. Previously, we have shown a reduced insulin sensitivity (with Isi-gly, a whole body/peripherical insulin sensitivity) in FF group [12] and higher glycaemia at time 0, 15 min for OGTT curves compared to C (102.1 ± 3.1 vs. 83.9 ± 1.6, at *T* = 0 and 149.0 ± 7.41 vs.126.3 ± 6.1 mg/dL). In our case, by using net glucose AUC for identifying IGT, it becomes possible to bring out AUC of the C group. For that matter, if total AUC is used rather than net AUC (data not shown), only training will improve IGT (reduced total glucose AUC). Consequently, as C, FF, and ExFF group are not statistically different, we propose to invalidate IGT in C group with net glucose AUC. Nonetheless, IGT is not systematically observed in fructose fed model of MetS, and this IGT would be dependent of gender [6], time of regimen [43, 44] and is improve with training [19].

An important point to discuss about MetS markers is the sex differences on the manifestation of MetS. In Sprague–Dawley rats, Sangüesa and colleagues (2018) [45] report that fructose induced hypertriglyceridemia and fatty liver in both sexes, but only females showed IGT and hepatic insulin resistance. Wistar strain rat is less sensitive towards MetS than Sprague-Dawley [12]. Using Wistar, Koricanac and colleagues (2013) [46] have also support the idea of gender-dependent differences in the expression of the metabolic syndrome phenotype with 10% of fructose solution in tap water. In fact, female rats were characterized by decreased glycemia, increased triglycerides, enlarged visceral adipose tissue and increased absolute mass of liver, without changes in systolic blood pressure and insulin sensitivity. In contrast, male rats developed less disturbances in physical and biochemical characteristics, but blood pressure and insulin sensitivity were impaired by fructose diet. In this paper, working only on male wistar rats makes sense to determine training effect on MetS development avoiding estrogen protector effects [47].

The first 6 weeks of fructose intake, rats are not in overweight. Previous reports showed that the overweight is not in the first MetS markers systematically associated to MetS development [11, 12, 43]. In our study, the overweight obtained with twelve weeks of fructose supplemented animal is reduced by ET at a level similar to exercise trained rats with a standard diet, without heart weight and total calorie intake differences (Fig. 1a and Table 3). Moreover, fructose diet caused stimulation of satiety center thereby reducing food intake [9]. Otherwise, higher heart weight FF group compared to control can be associated with a higher systolic blood pressure [48]. In our previous data [11], a similar fructose supplementation induced hypertension that is maintained for 21 weeks. Renal function is often affected in T2D cases [49] and can be linked to higher blood pressure. In this purpose, theoretical creatinine clearance was calculated and surprisingly the fructose enriched diet increased this marker thus indicating a better renal function. More surprisingly, exercise trained animals appear to have a lower renal function as they display a reduced theoretical creatinine clearance. This result is not consistent with the current knowledge, as plasma protein clearance is known to be increase with ET [50]. However, in our knowledge, no study on the modification of the theoretical creatinine clearance following ET was found. A possible explanation may be that theoretical creatinine clearance formula includes body weight in a position where lowering body weight decreases the clearance value. As exercise trained rats presented lower body weight than sedentary animals, it may explain our observation.

Many studies have concluded that plasma leptin and adiponectin are associated with obesity and MetS, and can be used as diagnostic markers for MetS [51]. The LAR may be useful to identify subjects susceptible to metabolic risk, and may reflect the functionality of adipose tissue [13]. Moreover, the presence of MetS leads to high LAR associated with an increasing number of MetS components [13, 15]. Frühbeck and colleagues (2017) [52] proposed that LAR performed in humans below 1 is considered as normal, ratio between 1 and 2 is in moderate cardiometabolic risk, and ratio above 2 is in severe cardiometabolic risk. Surprisingly, our study showed no difference between C and FF groups with LAR below 1. Consequently, these rats could be considered “metabolically healthy” or “normal” with a functional adipose tissue. Exercise has been reported to increase and reduce adiponectin and leptin concentrations, respectively, although a different impact may be produced depending on the type of exercise (endurance vs. resistance exercise) [53, 54]. Our personalized moderate endurance protocol shows that LAR is decreased with training about two-fold compared to untrained groups. Clearly, in the context of prevention of MetS, moderate exercise is efficient on LAR and some components of MetS like IR.

Previously, the liver was considered the primary site of sugar metabolism. Jang and colleagues (2018) [55] have shown, in C56BL/6 mice, that fructose at low dietary doses, does not reach the liver; instead, it is converted to glucose and other metabolites in the small intestine. But at higher doses, the intestine can not keep up, leaving the liver and bacteria of the colon to handle with the excess of fructose [55]. It is known that nearly all the ingested fructose is metabolized in the liver, the majority is converted into glucose, while around 15% is converted into glycogen, only a small proportion of which is converted into TG by de novo lipogenesis, usually around 1–5% [56]. However in case of fructose overload, like in our fructose supplemented rats, this percentage can rise up to 10% [57].

Even though dyslipidemia is known to be induced by fructose enriched diet in both human and Sprague-Dawley rats [58, 59], it does not systematically appear in Wistar rats [11, 60]. Neither ET nor fructose supplementation appeared to have an impact on NEFA levels. ET seems to have various effects depending on the diet. In Ex group only, higher total cholesterol is observed and in ExFF group, training does not reduce the TG level, which has increased with fructose-diet. Botezelli and colleagues (2010, 2016) [41, 61] also observed higher TG levels on fructose fed rats whatever length and modalities of training. During moderate intensity, lipids and carbohydrates are the preferential energy sources of muscular cells. In the case of fructose supplemented rats, adaptation to exercise may have promoted fructose oxidation and suppressed its hepatic accumulation [62]. As exercise decreases hepatic accumulation of TG from fructose fed, thus it is increasing circulating lipids availability [61]. An overload of fructose promotes de novo synthesis of TG: in our study, we could suppose that exercise does not reduce this mechanism. These circulating lipids are then used for energy production instead of being stored in the liver or adipose tissue. This idea is be comforted by the observation of Lozano and colleagues (2016) [43]: using similar fructose supplementation as ours, rats did not present increased hepatic TG levels even though their hepatic glycogen levels were higher. We can suppose that the TG stay in the plasma to be available for the metabolism.

Meanwhile, different molar ratios were calculated from the lipids levels results (Table 4). Total cholesterol/TG molar ratio is decreased with the fructose enriched diet and exercise alone improves it. This ratio predicts the presence of small density LDL both in human [35] and murine models [63]. The lower the ratio is, the higher the presence of small density LDL is and, as a consequence, the higher the cardiovascular risks are [64]. Consequently, if we have no explanation for the upper value of total cholesterol in Ex group compared to C and ExFF, training is in favor of reducing cardiovascular risk factors. We could suggest that our training protocol improve HDL compared to the others groups especially as the total plasma cholesterol is about 80% in HDL particles in rats because of the absence of plasma cholesteryl ester transfer protein [65]. In this paper, all the performed assays were plasmatic ones. We have not performed hepatic and adipose tissue TG measurements. The cholesterol pathway was not evaluated and we have only total cholesterol. Like Botezelli and colleagues (2016) [41], we found an increase of plasmatic triglyceride in ExFF without modification of total cholesterol. These authors observed an increase of HDL-cholesterol with training. We agree with them when they explain that improvement in liver function or high TG consumption may be due to aerobic exercise. We suppose the HDL cholesterol level is more enhanced with training without fructose fed. NEFA/Albumin is a parameter that indicating cardiovascular risks, particularly less coronary heart disease risk [34] in trained animals. In our study, no difference was found between the groups. According to these markers, moderate exercise is beneficial for the cardiovascular system whereas fructose enriched diet increased cardiovascular risks, even if moderate training was performed.

In order to study MetS markers often associated with T2D, such as liver dysfunction and dyslipidemia, further blood biochemistry analyses were performed (Table 4 and Fig. 2). Cutoff of NEFA (0.566 mM) is never joined in the four groups. The NEFA/cholesterol molar ratio, another indicator of a liver dysfunction [11, 12], is not modified by the experiments. This suggests there is no hepatic lipidosis that was confirmed by no steatosis, considered as the hepatic manifestation of the MetS. Hepatic steatosis is commonly diagnosed when 5% of hepatocytes contain large lipid droplets or when intrahepatic TG content is superior to 5.6%, but the definition of normal liver fat content depends on the assessment method [66]. In an other hand, blood marker of liver function can be the ASAT/ALAT ratio. A higher ASAT/ALAT ratio may, indeed, indicates a liver dysfunction [67]. Fructose enriched diet increase ASAT/ALAT ratio, however both the diet and the ET interact together. Indeed, in fructose fed rats, ET increases the ASAT/ALAT ratio whereas in healthy rat exercise decreases it. No study on this ratio was found on exercise trained rats however it is known that ET induces mainly higher ASAT levels [68] and sometimes ALAT level [69] in human. The increase is more intense just after exercise, ALAT levels are normalized in 48 h hours whereas ASAT levels are in 9 days [69]. This regulation period may be longer in fructose fed rats in which metabolism is modified as it induced MetS markers [12]. However no study on the regulation of ASAT levels in T2D rats was found and this hypothesis can thus not be confirmed.

## Conclusion

The present study examined the influence of ET on MetS markers. After 6 weeks, ET fought the negative effects of fructose-enriched diet. Our data provided evidences that ET reduced body weight in fructose fed rats and improved glucose metabolism. Indeed, hyperglycaemia and IR were reduced by enhancement of insulin muscular sensitivity. It was very likely that ET contributed to diminish the fructose fed adipose tissue dysfunction. Nevertheless, it is possible that the excess of TG de novo synthesis induced by fructose could not be counterbalanced by ET and would contribute to maintain hypertriglyceridemia. Machado and colleagues (2017) [70] recently proposed that frequency and duration of aerobic exercise are determinant factors for the reversal of metabolic disorders in the experimental model of MetS induced by fructose diet. Our results indicated that positive health effects were achieved through a personalized moderate training of 300 min per week (1 h/ day and 5 days a week) for 6 weeks. So, it is reasonable to assume that duration of our personalized training (6 weeks) must be higher to accentuate the positive effects of ET.

## Abbreviations

ALAT: Alanine aminotransferase activity
ASAT: Aspartate aminotransferase activity
AUC: Area Under the Curve
Bw: body weight
CS: Citrate Synthase
ET: exercise training
FW: Fresh Weight
HDL: High Density Lipoprotein
HIRI: Hepatic Insulin Resistance Index
HOMA 2-IR: Homeostatic Model Assessment 2 for Insulin Resistance
IGT: Impaired Glucose tolerance
IR: insulin resistance
LAR: Leptin/Adiponectin ratio
LDL: Low Density Lipoprotein
MAS: maximal aerobic speed
MetS: Metabolic Syndrom
MISI: Muscle Insulin Sensitivity Index
NEFA: Non-esterified fatty acid
NS: Non Significant
OGTT: Oral Glucose Tolerance Test
SEM: standard error of mean
T2D: Type 2 Diabetes
TG: triglycerides
